# Food Safety, Hygiene, and Sanitation Practices Among University Campus Dining Personnel: An Institution‐Based Cross‐Sectional Study

**DOI:** 10.1155/jnme/7216438

**Published:** 2026-01-30

**Authors:** Md. Rakibul Hasan, Apurba Chowhan, Most. Ruksana Khatun, Mst. Mariam Sultana Saema, Tanjina Haque, Somaiya Islam Shuchy, Sunamdeb Chandra Pramanik, Farhana Akther

**Affiliations:** ^1^ Department of Food Technology and Nutritional Science, Mawlana Bhashani Science and Technology University, Santosh, Tangail, Bangladesh, mbstu.ac.bd; ^2^ Department of Nutrition and Food Engineering, Daffodil International University, Savar, Dhaka, Bangladesh, daffodilvarsity.edu.bd; ^3^ Department of Statistics, Mawlana Bhashani Science and Technology University, Santosh, Tangail, Bangladesh, mbstu.ac.bd

**Keywords:** attitude, food handlers, food safety, knowledge, practices

## Abstract

Foodborne illnesses are a major global health issue, often due to poor food safety practices. The specific aim of this study was to assess the level of knowledge, attitude, and practices (KAPs) and to identify the discrepancies between self‐reported and actual food safety practices among campus dining personnel. A cross‐sectional survey among 70 food handlers from 9 food outlets was conducted to collect self‐reported data on KAPs regarding hygiene, food handling, and safety practices using a structured questionnaire. About 90% of food handlers claimed adherence to hygiene protocols, no handlers were observed wearing aprons or hairnets, and only 11.1% used disposable gloves. Although 77.8% reported regular hand washing, only one outlet (11.1%) had functional hand washing facilities. Unsafe thawing methods were observed in all outlets, with 88.9% failing to use gloves for ready‐to‐eat foods. For practices, knowledge emerged as a significant predictor (*p* < 0.05), with an odds ratio of 0.353. Inadequate practices extended to food preparation, where only 11.1% used separate cutting boards for raw and cooked foods. Storage conditions were generally better, with 100% maintaining cold storage at < 5 °C, and 77.8% ensuring the separation of raw and cooked foods. The logistic regression analysis demonstrated a significant association (*p* < 0.01) between knowledge and attitudes and an odds ratio of 0.105 (CI: 0.035–0.315). Conversely, education showed no significant association with attitudes (*p* > 0.05; CI: 0.482–4.346). For practices, knowledge emerged as a significant predictor (*p* < 0.05), with an odds ratio of 0.353 (CI: 0.132–0.942). These findings reveal a critical gap between knowledge and application of food safety practices, attributed to infrastructure deficiencies and a lack of enforcement.

## 1. Introduction

Food, as a basic human necessity, plays a critical role in sustaining life, yet it is also a frequent source of diseases that can lead to significant morbidity and mortality worldwide [1]. Food is a highly susceptible medium for illness‐causing microbes, as impurities can come from a range of sources, such as water, air, equipment, and contact with humans [2]. There are approximately about 600 million cases of infections caused by food worldwide each year, according to the World Health Organization, and 31 foodborne agents are responsible for 420,000 deaths [3]. In Bangladesh, foodborne diseases are a significant public health concern. On an annual basis, about 30 million people contract foodborne illnesses; the most frequent causes are *Salmonella*, *Shigella*, *Escherichia coli*, Rotavirus, and *Vibrio cholerae* [4]. The country’s high rate of foodborne illnesses emphasizes just how important it is to be aware of the causes of food contamination and sickness, especially in the food establishment. Food establishments, including restaurants, cafeterias, and dining halls, are key points in the food supply chain where contamination can occur. According to the Centers for Disease Control and Prevention (CDC), 59% of foodborne disease outbreaks are linked to these establishments, with improper food handling practices by employees being a major cause [5]. Food handlers play a crucial role in preventing contamination; however, their knowledge, attitudes, and practices (KAPs) regarding food hygiene and sanitation are often inadequate. Research indicates that over 75% of foodborne illness outbreaks can be traced back to poor food handling practices, such as improper food storage, inadequate cooking temperatures, and poor personal hygiene [2]. In Bangladesh, preparing, serving, and consuming food with bare hands is common [6]. For instance, a National Hygiene Survey in 2018 revealed that only 23% of food handlers in Bangladesh washed their hands with soap and water before preparing food, highlighting a critical gap in hygiene practices [7]. Moreover, to ensure food safety across the country, the Government of Bangladesh enacted the National Food Safety Act 2013, which makes it mandatory for all food manufacturers, processors, and handlers to comply with established food safety and hygiene standards [8]. This legislation serves as a legal framework to safeguard public health and underscores the responsibility of food handlers in maintaining safe practices. The food handlers play a significant role in preventing foodborne infections, particularly in settings like campuses where a lot of students eat food that has been prepared by others. Food poisoning can have severe consequences for students, affecting their health, academic performance, and overall well‐being. Moreover, food poisoning can lead to long‐term health effects, such as meningitis, kidney damage, and arthritis, further highlighting the importance of proper food hygiene and sanitation [9]. This study was conducted to provide evidence on the current food hygiene and sanitation KAP among university food handlers, identify gaps that could contribute to foodborne illnesses, and highlight areas for targeted interventions to improve food safety and protect public health. Additionally, the study will also explore the relationship between the food handler’s sociodemographic characteristics and their KAP levels, aiming to identify specific factors that may influence food safety practices.

## 2. Materials and Methods

### 2.1. Study Design and Sample Size

A cross‐sectional study was conducted among the food handlers using a purposive sampling strategy working in the 9 food establishments (outlets), which comprised the cafeteria and seven residential halls and guest house at Mawlana Bhashani Science and Technology University in Tangail, Bangladesh. The sample size was determined based on the total number of food handlers available in the selected university campus dining establishments. Therefore, 70 food handlers present during the study period were included to ensure inclusion of all available personnel directly involved in food preparation and handling during the study period. Data were collected over a period of 3 months (July to September, 2024). The research team visited the food establishments during the daytime for data collection, observation, and interviews. The participants were not informed in advance about the visits, which minimized the risk of observation bias. Food handler’s practices, kitchen areas, food cooking, and storage areas were directly observed to investigate the gaps between self‐reported knowledge level and practices.

### 2.2. Inclusion and Exclusion Criteria

Food handlers employed in the food establishments within the university territory and directly involved in food preparation or handling were included in the study. Food handlers who were on extended leave during the data collection period or those not directly engaged in food handling activities were excluded from the study.

### 2.3. Data Collection Tool and Procedure

Data collection was conducted by face‐to‐face interviews using a structured questionnaire. Prior to the main survey, the questionnaire was pretested to ensure clarity and relevance. After receiving approval from the relevant authorities, the participants were provided with a brief explanation of the study, and informed consent was obtained. The questionnaires were divided into two sections: One was the food safety, hygiene, and sanitation KAP, and another was the demographic information. Furthermore, on the KAP section, there were 3 parts: knowledge, attitude, and practices. The knowledge and attitude sections each featured 18 questions, while the practice section contained 20 questions. The questionnaire was designed following Abdul‐Mutalib et al. [10] and Halim‐Lim et al. [11]. The questionnaire was translated into Bangla for better understanding of the respondents. Four interviewers used a qualitative method, observing how food was handled, according to the method described by Halim‐Lim et al. [11]. The questionnaire has been provided as Supporting material.

#### 2.3.1. Assessment of KAP

Knowledge section of the questionnaire contained 17 structured questions on the knowledge level with 2 likely responses: “Yes” and “No.” The questions precisely covered the respondents’ knowledge of food safety, individual cleanliness, foodborne illnesses, microbes, infection control, and sanitary practices.

### 2.4. Ethical Consideration

Ethical clearance was obtained from Dept. of Food Technology and Nutritional Science, Mawlana Bhashani Science and Technology University, Tangail, Bangladesh, for data collection. The study received formal approval from the Ethical Review Committee of the Department of Food Technology and Nutritional Science under approval number FTNS/ERC/13/2024. Written informed consent was obtained from participants.

### 2.5. Data Analysis

Coding and analysis were done in IBM Statistical Package for Social Sciences (SPSS Inc., Chicago, USA; https://www.spss.com), Version 24.0. Following Rabeya et al. [12], each correct knowledge item reported was awarded a score of 1 point, and incorrect knowledge was awarded a 0 score. Attitude queries relating to attitudes in the third segment of the questionnaire contain 18 structured queries with 3 likely answers: “agree,” “disagree,” and “uncertain.” Each correct attitude reported was awarded a score of 1 point, while the other incorrect attitude option was rated a 0 score. In this study, if “agree” is the correct response, it receives a score of 1 point, whereas “disagree” receives 0 points or vice versa. Practice section of the questionnaire also contained 20 structured queries with 4 likely answers: “always,” “sometimes,” “often,” and “never” marking 3, 2, 1, and 0, respectively, when “always” is the correct answer. When “Never” is the correct answer, the score goes reverse vice versa. To categorize, following Abdul‐Mutalib et al. [10] for both the knowledge and attitude parts, the correct answers were converted to 100% and 50%–74.99% was considered acceptable knowledge and attitude, and marks 75% and above were considered excellent knowledge and attitude. The marks were converted to poor (marks below 50%) and good practice (50% and above). Categorical variables were expressed as frequency and percentage. The disparity between categorical variable groups was verified using the chi‐square test where appropriate. A *p* value < 0.05 was considered statistically significant.

## 3. Results

A total of 70 food handlers were invited and all 70 participated in the study, yielding a response rate of 100%.

### 3.1. Sociodemographic Characteristics

The sociodemographic characteristics of the food handlers are presented in Table 1. The gender distribution was nearly balanced, with 51.42% female and 48.58% male participants. Most participants (95.7%) were Muslim, with a minority (4.3%) identifying as Hindu. Assistant cooks made up the largest proportion (47.1%), followed by table attendants (34.3%), cooks (15.7%), and counter attendants (2.9%). Age‐wise, 60% of participants were over 30 years, suggesting a workforce with potentially more experience and maturity in handling food safely. Educational levels showed a slight majority (52.9%) with lower qualifications. About 30% had less than one year of experience, while 27.1% had been in the field for 11–15 years.

**Table 1:** Sociodemographic characteristics.

Variables		*n* (%)
Gender	Male	34 (48.58%)
	Female	36 (51.42%)

Religion	Islam	67 (95.7%)
	Hindu	3 (4.3%)

Designation	Cook	11 (15.7%)
	Assistant Cook	33 (47.1%)
	Table Boy/Girl	24 (34.3%)
	Counter Attendant	2 (2.9%)

Age group	≤ 30	28 (40%)
	> 30	42 (60%)

Educational qualification	Lower (below primary)	37 (52.9%)
	Higher (above primary)	33 (47.1%)

Working experience	< 1 year	21 (30%)
	1–5 years	10 (14.3%)
	6–10 years	18 (25.7%)
	11–15 years	19 (27.1%)
	16–20 years	2 (2.9%)

### 3.2. Knowledge of Food Handlers

Table 2 reveals that a significant 58.6% of participants demonstrated awareness of food hygiene and sanitation principles, which reflects a baseline understanding. While all respondents recognized the importance of hand washing before and after preparing food, using the bathroom, and handling waste (100%), only 47.1% understood the risks associated with wearing jewelry during food preparation. The importance of separating raw and cooked foods was well understood by 94.3% of respondents. However, only 51.4% knew that the same cutting board should not be used for raw vegetables and meats. Similarly, only 72.9% of respondents were aware that raw meat should be stored on the bottom shelf of the refrigerator to prevent drips from contaminating other foods.

**Table 2:** Respondents′ knowledge about food hygiene and sanitation.

Statements	Yes (%)	No (%)
Having knowledge about food hygiene and sanitation.	**41 (58.6%)**	29 (41.4%)
Wash hands before and after preparing food, using the bathroom, and clearing tables.	**70 (100%)**	0 (0%)
Jewelry should not be worn by a food handler while preparing food, as it can contain dirt and pathogens.	**33 (47.1%)**	37 (52.9%)
It is important to wash hands after handling waste.	**70 (100%)**	0 (0%)
Money is dirty and has the potential to transmit germs	**61 (87.1%)**	9 (12.9%)
Wiping clothes has the potential to transmit germs and lead to illness.	**62 (88.6%)**	8 (11.4%)
An uncooked meal should be stored separately from the prepared meal.	**66 (94.3%)**	4 (5.7%)
The same cutting board can be used for both raw vegetables and meats.	36 (51.4%)	**34 (48.6%)**
The best place to store raw fish, chicken, and meat in the refrigerator is the bottom shelf.	**51 (72.9%)**	19 (27.1%)
The surface that comes into contact with food must be cleaned with soap and water and then sanitized.	**50 (71.4%)**	20 (28.6%)
The ideal method to defrost meat is by keeping the meat on a dry countertop.	12 (17.1%)	**58 (82.9%)**
The ideal temperature for storing easily spoiled food, for example, vegetables and fruits, is 10 °C.	**16 (22.9%)**	54 (77.1%)
Hot, ready‐to‐eat food must be kept above the temperature of 60 °C.	**8 (11.4%)**	62 (88.6%)
An excess meal should be kept at the zone temperature and eaten for the following mealtime.	21 (30%)	**49 (70%)**
The refrigerator temperature should be within 5 °C–10 °C.	17 (24.3%)	**53 (75.7%)**
The best temperature for the growth of foodborne microorganisms is between 5 °C and 60 °C.	**14 (20%)**	56 (80%)
*Salmonella*, *Shigella*, Hepatitis A virus, and *Staphylococcus aureus* are associated with foodborne illnesses.	**30 (42.9%)**	40 (57.1%)

*Note:* Bold numbers indicate the correct knowledge responses.

The knowledge about temperature control and storage practices also exhibited significant deficiencies. About 17.1% of respondents stated that defrosting meat on a dry countertop is inappropriate, while only 11.4% understood that hot, ready‐to‐eat food must be kept above 60 °C to prevent microbial growth. Furthermore, misconceptions about refrigerator temperature were prevalent, with only 24.3% identifying the ideal range of 0 °C–4 °C. Only 20% knew the temperature range most conducive to microbial growth (5 °C–60 °C). Moreover, only 42.9% of respondents could identify key pathogens associated with foodborne illnesses, such as *Salmonella* and *Shigella*.

### 3.3. Attitudes of Food Handlers

The findings in Table 3 reveal a generally positive attitude among respondents toward food hygiene and sanitation. All respondents agreed on the necessity of cleaning work areas and washing hands before starting work, as well as maintaining proper hygienic habits. However, discrepancies in attitudes toward other critical aspects of hygiene were evident, indicating areas that require intervention. For instance, while 80% of respondents agreed on covering their mouth and nose when coughing or sneezing, a notable 15.7% were uncertain, and 4.3% disagreed. This behavior is crucial for minimizing the spread of respiratory pathogens in food settings. Similarly, while 85.7% opposed smoking while working, 8.6% disagreed. Attitudes toward avoiding contact between hands and the face, hair, and other surfaces while working showed a split, with only 48.6% in agreement and 44.3% uncertain. Moreover, attitudes toward the use of separate towels for cleaning various surfaces were divided, with 48.6% agreeing and 30% disagreeing.

**Table 3:** Respondents’ attitudes about food hygiene and sanitation.

Statements	Agree	Disagree	Uncertain
Work area must be cleaned before start working	**70 (100%)**	0	0
Hands should be washed before start working	**70 (100%)**	0	0
It is very important for the employee to have proper hygienic habits	**70 (100%)**	0	0
I must cover my mouth and nose when coughing or sneezing	**56 (80%)**	3 (4.3%)	11 (15.7%)
I should not smoke while working	**60 (85.7%)**	6 (8.6%)	4 (5.7%)
I should not rub my hands on face, hair, etc., while working	**34 (48.6%)**	5 (7.1%)	31 (44.3%)
Same towel can be used to clean many places	34 (48.6%)	**21 (30%)**	15 (21.4%)
Separate kitchen utensils must be used to prepare raw and cooked foods	**50 (71.4%)**	6 (8.6%)	14 (20%)
Food should not be touched with a wounded hand	**33 (47.1%)**	13 (18.6%)	24 (34.3%)
I am willing to learn about the basics of food hygiene and safety	**69 (98.6%)**	0	1 (1.4%)
I think restaurant managers should organize advanced training such as hazard analysis critical control point for food handlers	**35 (50%)**	2 (2.9%)	33 (47.1)
I will inform my supervisor if I have diarrhea, wounds, or cuts	**62 (88.6%)**	6 (8.6%)	2 (2.9%)
I will take sick leave if I have diarrhea, wounds or cuts	**60 (85.7%)**	4 (5.7%)	6 (8.6%)
I will try to do my level best to always observe proper cleaning procedures	**70 (100%)**	0	0
Preventing food contamination and spoilage is my key responsibility	**66 (94.3%)**	4 (5.7%)	0
Expired food should never be consumed	**70 (100%)**	0	0
I consistently use gloves to handle non‐packed food even if my supervisor is absent	**39 (55.7%)**	19 (27.1%)	12 (17.1%)
It is my responsibility to offer safe, hygienic foods to customers	**67 (95.7%)**	2 (2.9%)	1 (1.4%)

*Note:* Bold numbers indicate the positive attitudes.

In terms of kitchen practices, 71.4% agreed on the importance of using separate utensils for raw and cooked foods. However, only 47.1% believed that food should not be handled with wounded hands. Furthermore, 98.6% expressed a willingness to learn about food hygiene basics, suggesting that targeted training programs could yield significant improvements. The findings also reveal that half of the respondents (50%) supported advanced training, such as hazard analysis critical control point (HACCP) systems.

### 3.4. Practices of Food Handlers

The results in Table 4 reveal both commendable practices and critical gaps in food hygiene and sanitation among respondents. Encouragingly, the majority consistently adhered to essential practices such as washing hands with soap and water before preparing food (94.3%) and after using the bathroom (95.7%), cleaning the work area before starting work (97.1%), and washing fruits and vegetables before slicing them (85.7%). Alarmingly, 68.6% of respondents reported never wearing gloves while handling food, and 64.3% worked with lesions or wounds without sufficient precautions, although 64.3% wore gloves when handling food with wounds. Furthermore, 81.4% admitted to unsafe practicing of handling money and food simultaneously.

**Table 4:** Respondents’ practices about food hygiene and sanitation.

Statements	Never	Always
I wear gloves when I handle food	48 (68.6%)	**22 (31.4%)**
I handle money and food at the same time	**57 (81.4%)**	13 (18.6%)
I wash my hands with water and soap before preparing food	4 (5.7%)	**64 (94.3%)**
I wash hands after using the bathroom	3 (4.3%)	**67 (95.7%)**
I work when I have diarrhea	**42 (60%)**	28 (40%)
I work when I have lesions/wounds on my hand	**25 (35.7%)**	45 (64.3%)
I allow my fingernails to grow	**13 (18.6%)**	57 (81.4%)
I wash vegetables and fruits before slicing them	10 (14.3%)	**60 (85.7%)**
I keep cooked meat or chicken at room temperature for more than 6 h	**26 (37.1%)**	44 (62.9%)
I clean the work area before starting work	2 (2.9%)	**68 (97.1%)**
I use a tissue when I am coughing or sneezing	12 (17.1%)	**58 (82.9%)**
I use my apron as a towel to clean my hand	**57 (81.4%)**	13 (18.6%)
I smoke while working	**61 (87.1%)**	9 (12.9%)
I wear jewelry and a watch while working	**32 (45.7%)**	38 (54.3%)
I separate raw food from cooked food	13 (18.6%)	**57 (81.4%)**
I use separate kitchen utensils (e.g., knives, spoons) to prepare raw and cooked foods	13 (18.6%)	**57 (81.4%)**
When I have wounds on my hands, I wear gloves	45 (64.3%)	**25 (35.7%)**
I cover my hair with a cap or hairnet while handling or cooking food	52 (74.3%)	**18 (25.7%)**
I read the instructions on the storage of packaged food	22 (31.4%)	**48 (68.6%)**
I control pests.	10 (14.3%)	**60 (85.7%)**

*Note:* Bold numbers indicate the positive practices.

About 60% of respondents continued working while experiencing diarrhea. Additionally, while 81.4% consistently separated raw from cooked food and used separate utensils, the remaining 18.6% failed to adhere to this critical preventive measure against cross‐contamination. Although 74.3% covered their hair with caps or hairnets and 82.9% used tissues when coughing or sneezing, a notable minority neglected these essential measures. Moreover, only 68.6% read storage instructions for packaged foods, and 37.1% kept cooked meat or chicken at room temperature for over 6 hours. The results also highlighted that 45.7% wore jewelry or watches while working, and only 14.3% actively controlled pests.

### 3.5. Association of KAP and Sociodemographic Variables

Table 5 presents the associations between KAP levels and the sociodemographic characteristics of respondents. The association between gender and knowledge level approached significance (*p* > 0.05), with more males (58.8%) reporting an excellent knowledge level compared to females (36.1%). Age group showed no significant association with knowledge level (*p* > 0.05), although respondents over 30 years old exhibited higher levels of excellent knowledge (60%) than those aged 30 or younger (39.3%).

**TABLE 5:** Association between KAP and respondents′ sociodemographic characteristics.

Variables		Knowledge level		*n* (%)	χ^2^	*p* value
		Acceptable	Excellent			
Gender	Male	14 (41.2%)	20 (58.8%)	34 (48.58%)	3.620	0.057
	Female	23 (63.9%)	13 (36.1%)	36 (51.42%)		

Age group	≤ 30	11 (39.3%)	17 (60.7%)	28 (40%)	3.449	0.063
	> 30	26 (61.9%)	16 (38.1%)	42 (60%)		

Educational qualification	Lower	21 (56.8%)	16 (43.2%)	37 (52.9%)	0.479	0.489
	Higher	16 (48.5%)	17 (51.5%)	33 (47.1%)		

Working since food establishment	< 12 months	8 (38.1%)	13 (61.9%)	21 (30%)	3.268	0.514
	1–5 years	7 (70%)	3 (30%)	10 (14.3%)		
	6–10 years	10 (55.6%)	8 (44.4%)	18 (25.7%)		
	11–15 years	11 (57.9%)	8 (42.1%)	19 (27.1%)		
	16–20 years	1 (50%)	1 (50%)	2 (2.9%)		

**Variables**		**Attitude level**		***n* (%)**	**χ** ^2^	**p** **value**
		**Acceptable**	**Excellent**			

Gender	Male	16 (47.1%)	18 (52.9%)	34 (48.58%)	1.391	0.238
	Female	22 (61.1%)	14 (38.9%)	36 (51.42%)		

Age group	≤ 30	15 (53.6%)	13 (46.4%)	28 (40%)	0.010	0.922
	> 30	23 (54.8%)	19 (45.2%)	42 (60%)		

Educational qualification	Lower	22 (59.5%)	15 (40.5%)	37 (52.9%)	0.847	0.358
	Higher	16 (48.5%)	17 (51.5%)	33 (47.1%)		

Working since food establishment	< 12 months	9 (42.9%)	12 (57.1%)	21 (30%)	5.153	0.272
	1–5 years	6 (60%)	4 (40%)	10 (14.3%)		
	6–10 years	8 (44.4%)	10 (55.6%)	18 (25.7%)		
	11–15 years	13 (68.4%)	6 (31.6%)	19 (27.1%)		
	16–20 years	2 (100%)	0 (0%)	2 (2.9%)		

Knowledge level	Acceptable	24 (75.0%)	9 (23.7%)	33 (47.1%)	18.358	< 0.001
	Excellent	8 (25.0%)	29 (76.3%	37 (52.9%)		

**Variables**		**Practice level**		***n* (%)**	**χ** ^2^	**p** **value**
		**Poor**	**Good**			

Gender	Male	13 (38.2%)	21 (61.8%)	34 (48.58%)	0.278	0.598
	Female	16 (44.4%)	20 (55.6%)	36 (51.42%)		

Age group	≤ 30	12 (42.9%)	16 (57.1%)	28 (40%)	0.039	0.843
	> 30	17 (40.5%)	25 (59.5%)	42 (60%)		

Educational qualification	Lower	17 (45.9%)	20 (54.1%)	37 (52.9%)	0.660	0.417
	Higher	12 (36.4%)	21 (63.6%)	33 (47.1%)		

Working since food establishment	< 12 months	12 (57.1%)	9 (42.9%)	21 (30%)	8.821	0.066
	1–5 years	3 (30%)	7 (70%)	10 (14.3%)		
	6–10 years	10 (55.6%)	8 (44.4%)	18 (25.7%)		
	11–15 years	4 (21.1%)	15 (78.9%)	19 (27.1%)		
	16–20 years	0 (0%)	2 (100%)	2 (2.9%)		

Knowledge level	Acceptable	18 (62.1%)	15 (36.6%)	33 (47.1%)	4.427	0.035
	Excellent	11 (37.9%)	26 (63.4%)	37 (52.9%)		

Attitude level	Acceptable	19 (65.5%)	13 (31.7%)	32 (45.7%)	7.824	0.005
	Excellent	10 (34.5%)	28 (68.3%)	38 (54.3%)		

Educational qualification did not show a significant effect on knowledge level (*p* > 0.05). Work experience was also not significantly associated with knowledge level (*p* > 0.05). The results also showed no significant association between gender and attitude level (*p* > 0.05), although females had a higher percentage (61.1%) of excellent attitudes than males (52.9%). Educational qualification was not significantly associated with attitude (*p* > 0.05). Work experience significantly influenced attitude, especially those with 16–20 years of experience who demonstrated perfect attitudes toward food hygiene. Knowledge and attitude levels were found to significantly correlate with practice levels (knowledge: χ^2^ = 4.427, *p* < 0.05; attitude: χ^2^ = 7.824, *p* < 0.01).

### 3.6. Logistic Regression Analysis

Table 6 exhibits the results of logistic regression analysis predicting food handlers′ attitudes and practices based on their knowledge and education levels. Knowledge emerged as a significant predictor of food handlers′ attitudes (Wald χ^2^ = 16.186, *p* < 0.01), with an odds ratio of 0.105 (95% CI: 0.035–0.315). In contrast, education level did not significantly predict attitude (Wald χ^2^ = 0.434, *p* > 0.05), with an odds ratio of 1.447, indicating no meaningful contribution of educational background to attitudes in this sample.

**Table 6:** Logistic regression predicting food handlers’ attitude and practices with variables.

Attitude versus education and knowledge					
Variables	Wald χ^2^	*p* value	EXP(B)	95.0% CI for EXP(B)	
				Lower	Upper
Knowledge	16.186	< 0.001	0.105	0.035	0.315
Education	0.434	0.510	1.447	0.482	4.346
Constant	4.843	0.028	2.967		

**Practice versus knowledge**					
**Variables**	**Wald** **χ** ^2^	***p* value**	**EXP (B)**	**95.0% CI for EXP(B)**	
				**Lower**	**Upper**

Knowledge	4.320	0.038	0.353	0.132	0.942
Constant	5.720	0.017	2.364		

Table 6 also examines that knowledge significantly predicted food hygiene practices (Wald χ^2^ = 4.320, *p* = 0.038), with an odds ratio of 0.353 (95% CI: 0.132–0.942), suggesting that higher knowledge levels increase the likelihood of adhering to proper practices.

### 3.7. Observational Analysis

Table 7 shows a detailed inspection analysis of food safety practices across nine food outlets inside the selected institution. None of the food handlers from any outlets wore aprons, hairnets, or maintained clean uniforms during work, contrasting starkly with standard food safety requirements. Additionally, 88.9% of food outlets maintained their handlers with clean fingernails, a positive observation. However, only 11.1% outlets provide disposable gloves. In 77.8% of the outlets, food handlers were found preparing foods with wearing jewelry, which may pose contamination risks. While detergent was provided at all outlets, functional hand washing facilities and hand‐drying options were found in only 11.1% of cases.

**Table 7:** Inspection analysis of the food safety practices among the food outlets (*n* = 9).

Inspection list	Statements	Yes	No
Personal hygiene	Wearing an apron during work	0	9 (100%)
	If yes, cleanliness of uniform	0	9 (100%)
	Use your apron as a towel to clean your hands	0	9 (100%)
	Keeping fingernails short and clean.	8 (88.9%)	1 (11.1%)
	Wearing disposable gloves.	1 (11.1%)	8 (88.9%)
	Wearing hairnet.	0	9 (100%)
	Wear jewelry while preparing food.	7 (77.8%)	2 (22.2%)
	Suffering from any disease (norovirus, Hepatitis A, *Salmonella*, Flu, *E. coli* infection)	2 (22.2%)	7 (77.8%)

Hand washing facilities	Detergent provided.	9 (100%)	0
	Hand‐drying facilities provided.	1 (11.1%)	8 (88.9%)
	Functional hand washing facilities	1 (11.1%)	8 (88.9%)

Thawing of meat/chicken/fish	Kept in a bowl of water.	9 (100%)	0
	Kept inside the sink without a running water supply	9 (100%)	0
	Kept in a tray/bowl without water	3 (33.3%)	6 (66.7%)
	Used microwave and chiller.	1 (11.1%)	8 (88.9%)

Food handling practices	Use of gloves when handling ready‐to‐eat foods.	1 (11.1%)	8 (88.9%)
	Avoiding bare‐hand contact with food	0	9 (100%)
	Proper covering and storage of leftover food	2 (22.2%)	7 (77.8%)

Food preparation	Use of separate cutting boards for raw meats and other foods	1 (11.1%)	8 (88.9%)
	Proper cooking temperatures reached and maintained.	4 (44.4%)	5 (55.6%)
	Thawing of frozen foods done safely (e.g., not at room temperature).	4 (44.4%)	5 (55.6%)
	Reuse of same oil in cooking food.	6 (66.7%)	3 (33.3%)

Food storage	Cold at < 5 °C	9 (100%)	0
	Freeze at < −18 °C	6 (66.7%)	3 (33.3%)
	Hot food above 60 °C	7 (77.8%)	2 (22.2%)
	Raw and cooked foods are stored separately in the refrigerator.	7 (77.8%)	2 (22.2%)

Food display and service	Proper covering and protection of displayed food items to prevent contamination.	9 (100%)	0
	Regular cleaning and sanitizing of display cases, buffets, and serving utensils	2 (22.2%)	7 (77.8%)
	Regular checking of expiry date of food items and properly disposing the expired food items.	1 (11.1%)	8 (88.9%)

Wipe clothes	Cloth dirty/bad‐smelling	3 (33.3%)	6 (66.7%)
	Same clothes used.	8 (88.9%)	1 (11.1%)

Cleaning and sanitizing	Regular cleaning schedules for equipment, utensils, and surfaces	3 (33.3%)	6 (66.7%)
	Use of approved sanitizers for disinfecting surfaces.	1 (11.1%)	8 (88.9%)

Pest control	No signs of pests such as rodents, insects, or birds.	2 (22.2%)	7 (77.8%)
	Proper storage of food to prevent pest access.	0	9 (100%)

Waste management	Proper disposal of waste, including food scraps and packaging	3 (33.3%)	6 (66.7%)
	Adequate trash bins with lids to prevent contamination and odor.	0	9 (100%)
	Regular removal of waste to prevent buildup	9 (100%)	0

Unsafe thawing methods were prevalent, with 100% of outlets using bowls of water or sinks without running water, and only one outlet (11.1%) using safer methods like microwaves or chillers. A concerning observation was the complete absence of bare‐hand contact avoidance, highlighting serious hygiene lapses. Furthermore, only 22.2% of outlets stored leftovers properly, and only 11.1% used separate cutting boards for raw and cooked foods. Proper cooking temperatures and safe thawing of frozen foods were achieved in only 44.4% of outlets. Food storage practices showed mixed results. While all outlets maintained cold storage below 5 °C, only 66.7% achieved freezing below −18 °C. Most outlets (77.8%) stored raw and cooked foods separately, reducing contamination risks. However, food display practices were suboptimal, with only 22.2% regularly cleaning display cases or disposing of expired food items. Cleaning schedules were poorly implemented, with only 33.3% adhering to regular cleaning and using approved sanitizers. The use of dirty or bad‐smelling clothes was observed in 33.3% of outlets, while 88.9% reused cleaning clothes, a clear breach of sanitation standards. Pest control was inadequate, with signs of pests in 77.8% of outlets and improper food storage allowing pest access in all outlets. Waste management practices were equally poor; while waste was regularly removed, none of the outlets used trash bins with lids, increasing the risk of contamination and odors.

## 4. Discussion

The findings indicate that while food handlers possessed a basic awareness of hygiene and sanitation, their knowledge was fragmented, with significant weaknesses in technical areas, such as temperature control, cross‐contamination, and pathogen identification. This reflects a common trend observed in food safety research: General concepts are often understood, but detailed operational knowledge remains limited [13, 14]. The strong awareness of hand hygiene aligns with reports from Ethiopia and Malaysia, where promotional campaigns and public health messaging have reinforced this practice [15, 16]. However, less visible risks, such as contamination from jewelry or improper use of cutting boards, were poorly recognized. Similar oversights have been documented among food handlers in Ghana and Brazil, where personal hygiene practices are undervalued despite being potential vectors of microbial transfer [17, 18]. Deficiencies in temperature‐related knowledge are particularly concerning, as improper cooking, storage, and holding temperatures are among the leading causes of foodborne outbreaks in institutional settings. Evidence from studies in Saudi Arabia and Bangladesh highlights this gap as a persistent global issue, often linked to inadequate training and limited access to thermometers [14, 19]. Likewise, the low recognition of foodborne pathogens suggests that food handlers may lack an understanding of the microbiological basis of contamination, which reduces their ability to link unsafe practices to health outcomes. Overall, these findings underscore the need for structured, practical training that emphasizes not only general hygiene but also critical technical aspects of food safety. Reinforcement through supervision, refresher courses, and provision of basic tools could help translate knowledge into safer practices, thereby reducing outbreak risks in university food establishments.

The generally positive attitudes observed among food handlers suggest an openness to maintaining hygienic environments and a recognition of their responsibility for food safety. Such findings are encouraging, as attitude is often a strong predictor of compliance with safe practices when supported by adequate supervision and resources [13, 16]. Nonetheless, inconsistencies in certain domains—such as reluctance to consistently cover the mouth and nose when sneezing, uncertainty about avoiding hand contact with the face and hair, and divided opinions on towel use—indicate that not all handlers internalize the broader implications of hygiene lapses. Similar patterns have been documented in Ghana and Brazil, where gaps between ideal attitudes and actual workplace behaviors were attributed to insufficient reinforcement and limited risk perception [17, 18]. These findings highlight that while respondents endorsed obvious hygienic actions, subtler behaviors with indirect, but significant consequences for contamination risk were undervalued. Attitudes toward reporting illness and avoiding food handling while wounded were also mixed, reflecting challenges noted in other institutional settings where economic pressures and fear of job loss discourage disclosure [20]. This is critical, as food handlers with gastrointestinal symptoms are well‐established sources of outbreaks. The strong willingness to learn about food hygiene and the support expressed for advanced training, including HACCP, are promising. Previous studies have shown that positive attitudes toward training are associated with improved long‐term compliance when training is practical and repeated [21]. Leveraging this motivation through structured education and supervisory feedback could bridge the gap between knowledge, attitudes, and consistent practice in university dining environments.

The findings on food hygiene and sanitation practices illustrate a mixture of commendable habits and areas of serious concern. On the positive side, most food handlers demonstrated strong compliance with fundamental hygiene behaviors, including proper handwashing before food preparation and after using the toilet, cleaning work areas prior to use, and washing fresh produce before cutting. These practices indicate a baseline awareness of essential food safety measures. However, several critical gaps persist that could compromise food safety. A large proportion of food handlers reported not wearing gloves while handling food and continued working despite having wounds or skin lesions, which increases the risk of direct contamination. Even more concerning, many admitted to handling both food and money simultaneously—an unsafe practice that can facilitate microbial transfer. Similarly, a significant proportion continued working while experiencing diarrhea, reflecting a limited understanding of the relationship between personal health and foodborne disease transmission. Other risky behaviors included leaving cooked meat or chicken at room temperature for extended periods, wearing jewelry or watches during food handling, and neglecting pest control measures. While some food handlers practiced safe separation of raw and cooked foods and used separate utensils to avoid cross‐contamination, a notable minority did not adhere to these preventive measures. Additionally, inconsistent use of hair coverings, limited attention to storage instructions, and occasional use of aprons as towels highlight lapses in maintaining sanitary conditions. These findings indicate the need for targeted training, stricter enforcement of hygiene policies, and continuous monitoring to bridge the gap between knowledge and actual practice.

Gender appeared to have a marginal influence on knowledge, with males generally demonstrating stronger knowledge than females, though this difference did not reach statistical significance. Age also showed no significant association, but older respondents tended to have higher levels of excellent knowledge compared to younger counterparts, which is in agreement with previous studies [22, 23]. Similarly, educational qualifications and years of work experience were not significantly associated with knowledge, suggesting that practical exposure and formal education alone may not guarantee improved understanding of food hygiene [24]. When examining attitudes, no significant associations were observed with gender, age, or education, though females displayed a somewhat more favorable attitude than males. Work experience showed a trend toward influence, with those having longer service—particularly individuals with extensive experience—exhibiting more positive attitudes toward food hygiene and sanitation, which is consistent with previous studies [25]. This may reflect the impact of accumulated workplace exposure and adaptation to hygienic norms over time. Significantly, food handlers who demonstrated higher knowledge and more positive attitudes were also more likely to engage in good hygiene practices. This emphasizes the interdependence of the three domains, highlighting that knowledge and attitudes directly shape hygienic behaviors. Strengthening educational and training interventions could therefore play a pivotal role in improving practical compliance with food safety standards.

Knowledge significantly predicted attitudes toward food hygiene, indicating that handlers with stronger knowledge were more likely to exhibit positive attitudes. By contrast, education level did not significantly influence attitudes, suggesting that formal education alone may not adequately translate into safer behavioral orientations without targeted food safety training. Similarly, knowledge was also a significant predictor of hygienic practices, reinforcing the interdependence of the KAP framework. These findings align with evidence from previous studies, which emphasize that practical knowledge gained through training and awareness programs is more influential than educational background in driving compliance with food safety standards [26, 27]. Collectively, the results underscore the importance of strengthening continuous, workplace‐specific food safety education as a strategy to foster both attitudinal change and consistent hygienic practices among food handlers. The observational findings reveal substantial gaps between recommended food safety standards and actual practices in the institutional food outlets. Critical deficiencies were noted in personal hygiene, including the absence of aprons, hairnets, and gloves, and widespread use of jewelry during food preparation. The comparison between self‐reported and observed practices highlights a significant discrepancy, suggesting overestimation of compliance by food handlers. While participants reported adherence to hygiene and safe food handling protocols, direct observation indicated widespread noncompliance. Such gaps are consistent with prior studies, which have reported that self‐reports often overestimate safe practices due to social desirability bias or lack of awareness of proper standards [28]. These findings highlight the need for not only targeted training to improve knowledge‐to‐practice translation but also infrastructural improvements and stronger enforcement mechanisms to ensure consistent adherence to food safety protocols.

Gender, age, education, and work experience showed limited influence on knowledge, attitudes, or practices, consistent with studies suggesting that sociodemographic variables alone are weak predictors of food safety behaviors. While males and older respondents tended to report slightly better knowledge, and longer work experience was linked to more positive attitudes, these associations were not statistically significant, indicating that demographic background is not the primary driver of food safety compliance. In contrast, both the association analysis and logistic regression clearly demonstrated the central role of knowledge. Higher knowledge was significantly associated with more positive attitudes and better hygienic practices, a finding that supports the well‐established KAP framework [29]. Importantly, formal education level did not significantly predict attitudes, highlighting that general education may not equip food handlers with the applied knowledge necessary for safe food handling. Instead, targeted, workplace‐specific food safety training appears more effective in shaping behavior. These findings reinforce the need for continuous capacity‐building programs focusing on practical knowledge to foster attitudinal change and improve compliance with hygiene and sanitation practices among food handlers.

## 5. Conclusion

The findings of this study are particularly important as they reveal significant gaps in food handlers’ KAPs regarding food hygiene and sanitation. Despite some awareness of basic hygiene principles, many respondents demonstrated unsafe practices, such as improper temperature control, inadequate hand hygiene in certain contexts, and limited understanding of cross‐contamination risks. These observations directly address the study’s primary questions on discrepancies between self‐reported knowledge and observed practices. These deficiencies can directly contribute to the occurrence of foodborne illnesses, which pose serious public health risks, particularly in settings like university campuses where large populations consume food prepared by others. By identifying these gaps, this study underscores the critical need for targeted interventions. In particular, we recommend the development and implementation of comprehensive training programs, including practical hands‐on workshops, to bridge the gap between knowledge and practice. Furthermore, the regular monitoring and enforcement of food safety protocols should be prioritized, alongside improvements in infrastructure, such as functional handwashing facilities and adequate storage conditions. Consequently, this research not only provides a snapshot of the current food hygiene scenario among university food handlers but also highlights actionable areas for intervention to improve overall food safety and protect public health.

## 6. Limitations

The purposive sampling approach may have introduced selection and volunteer bias, limiting the representativeness of the sample. As the study focused only on food handlers within one university setting, the findings may not be fully generalizable to food handlers in Tangail or broader contexts; however, they provide a useful insight into the food hygiene scenario in public universities in Bangladesh. Additionally, the study did not include microbiological or physicochemical analyses of food or surfaces, which could provide direct evidence of contamination or hazards; this represents a potential area for future research.

## Funding

No funding was received for this manuscript.

## Conflicts of Interest

The authors declare no conflicts of interest.

## Supporting Information

Supporting Materials. The questionnaire used for data collection in this survey is provided as Supporting Material to support transparency and reproducibility of the study.

## Supporting information


**Supporting Information** Additional supporting information can be found online in the Supporting Information section.

## Data Availability

The data that support the findings of this study are available from the corresponding author upon reasonable request.
