# Supplementary material for: Food Safety, Hygiene, and Sanitation Practices Among University Campus Dining Personnel: An Institution‐Based Cross‐Sectional Study
Source: J Nutr Metab. 2026 Jan 30;2026:7216438. doi: 10.1155/jnme/7216438 (PMC12859165; doi:10.1155/jnme/7216438)
Supplement: Supplementary file 1 — Supporting Information Additional supporting information can be found online in the Supporting Information section. [file JNME-2026-7216438-s001.pdf]

Supplementary Material: Questionnaire

Questionnaire on

"Food Safety, Hygiene and Sanitation Practices among University Campus Dining Personnel: An Institution-Based Cross-Sectional Study"

A) Background information

1. Name of the campus dining services:

2. Location of the campus dining services:

3. Name of the respondent:

4. Respondent's age:

i. ≤30
ii. >30

5. Respondent's sex:

i. Male
ii. Female

6. Respondent's religion:

i. Islam
ii. Hindu

7. Educational qualification of the Respondent:

i. Lower
ii. Higher

8. Position at the Food establishment:

9. Working since this Food establishment:

B) KAP about Food Hygiene and Sanitation

Knowledge-

1. Do you understand food hygiene and sanitation? a) yes b) no

2. Wash hands before and after preparing food, using bathrooms, and clearing tables.

a) yes b) no

3. Jewellery should not be worn by food handler while preparing food, as it can contain dirt and pathogens. a) yes b) no

4. It is important to wash hands after handling waste. a) yes b) no

5. Money is dirty and has the potential to transmit germs a) yes b) no

6. Wiping clothes has the potential to transmit germs and lead to illness. a) yes b) no

7. An uncooked meal should be stored separately from the prepared meal. a) yes b) no

8. The same cutting board can be used for both raw vegetables and meats. a) yes b) no

9. The best place to store raw fish, chicken, and meat in the refrigerator is the bottom shelf. a) yes b) no

10. The surface that comes into contact with food must be cleaned with soap and water and then sanitized. a) yes b) no

11. The ideal method to defrost meat is by keeping the meat on a dry countertop. a) yes b) no

12. The ideal temperature for storing easily spoiled food for example vegetables and fruits is 10°C. a) yes b) no

13. Hot, ready-to-eat food must be kept above the temperature of 60°C. a) yes b) no

14. An excess meal should be kept at zone temperature and eaten for the following mealtime. a) yes b) no

15. The refrigerator temperature should be within 5°C to 10°C. a) yes b) no

16. The best temperature for the growth of foodborne microorganisms is between 25°C to 60°C. a) yes b) no

17. Salmonella, Shigella, Hepatitis A virus, and Staphylococcus aureus are associated with foodborne illnesses. a) yes b) no

Attitudes

1. Do you think work area must be cleaned before start working?

(a) Agree; (b) Disagree; (c) Uncertain

2. Do you think hands should be washed before start working?

(a) Agree; (b) Disagree; (c) Uncertain

3. Do you think it is very important for the employee to have proper hygienic habits.

(a) Agree; (b) Disagree; (c) Uncertain

4. Do you think we must cover our mouth and nose when coughing or sneezing?

(a) Agree; (b) Disagree; (c) Uncertain

5. Do you think we should not smoke while working?

(a) Agree; (b) Disagree; (c) Uncertain

6. Do you think we should not rub our hands on face, hair, etc. while working?

(a) Agree; (b) Disagree; (c) Uncertain

7. Do you think same towel can be used to clean many places?

(a) Agree; (b) Disagree; (c) Uncertain

8. Separate kitchen utensils must be used to prepare raw and cooked food?

(a) Agree; (b) Disagree; (c) Uncertain

9. Food should not be touched with wounded hand?

(a) Agree; (b) Disagree; (c) Uncertain

10. I am willing to learn about the basics of food hygiene and safety.

(a) Agree; (b) Disagree; (c) Uncertain

11. I think restaurant managers should organize advanced training such as hazard analysis critical control point for food handlers.

(a) Agree; (b) Disagree; (c) Uncertain

12. I will inform my supervisor if l have diarrhoea, wounds or cuts.

(a) Agree; (b) Disagree; (c) Uncertain

13. I will take sick leave if l have diarrhoea, wounds or cuts.

(a) Agree; (b) Disagree; (c) Uncertain

14. I will try to do my level best to always observe proper cleaning procedures.

(a) Agree; (b) Disagree; (c) Uncertain

15. Preventing food contamination and spoilage is my key responsibility

(a) Agree; (b) Disagree; (c) Uncertain

16. Expired food should never be consumed.

(a) Agree; (b) Disagree; (c) Uncertain

17. I consistently use gloves to handle non-packed food even if my supervisor is absent.

(a) Agree; (b) Disagree; (c) Uncertain

18. It is my responsibility to offer safe hygienic foods to customers.

(a) Agree; (b) Disagree; (c) Uncertain

Practice-

1. Do you wear gloves when you handle food?

a. Never b. Always

2. Do you handle money and food at the same time?

a. Never b. Always

3. Do you Wash hand with water and soap before preparing food?

a. Never b. Always

4. Do you Wash hand after using bathroom?

a. Never b. Always

5. Do you work when you have diarrhoea?

a. Never b. Always

6. Do you work when you have lesions /wound on your hand?

a. Never b. Always

7. Do you allow your fingernails to grow?

a. Never   b. Always

8. Do you wash vegetables and fruits before slicing them?

a. Never   b. Always

9. Do you keep cooked meat or chicken at room temp for more than 6 hours?

a. Never   b. Always

10. Do you clean the work area before starting work?

a. Never   b. Always

11. Do you use a tissue when you are coughing or sneezing?

a. Never   b. Always

12. Do you use your apron as a towel to clean your hand?

a. Never   b. Always

13. Do you smoke while working?

a. Never   b. Always

14. Do you wear jewellery and a watch while working?

a. Never   b. Always

15. Do you separate raw food from cooked food?

a. Never   b. Always

16. Do you use separate kitchen utensils (e.g., knives, spoons) to prepare raw and cooked food?

a. Never   b. Always

17. If you have wounds on your hand, do you wear gloves?

a. Never   b. Always

18. Do you cover your hair with a cap or hairnet while handling, or cooking food?

a. Never   b. Always

19. Have you read the instructions on the storage of packaged food?

a. Never   b. Always

20. How often do you control pests?

a. Never   b. Always

C) Observations by the enumerators:

Personal hygiene

- ✓ Wearing an apron during work. a) yes b) no
- ✓ If yes, Cleanliness of uniform. a) yes b) no
- ✓ Use your apron as a towel to clean your hands. a) yes b) no
- ✓ Keeping fingernails short and clean. a) yes b) no
- ✓ Wearing disposable gloves. a) yes b) no
- ✓ Wearing hairnet. a) yes b) no
- ✓ Don't wear jewellery while preparing food. a) yes b) no
- ✓ Suffering from any disease (Norovirus, Hepatitis A, Salmonella, Flu, E. coli infection). a) yes b) no

Handwashing facilities

- ✓ Detergent provided. a) yes b) no
- ✓ Hand drying facilities provided. a) yes b) no
- ✓ Functional handwashing facilities. a) yes b) no

Thawing of meat /chicken /fish

- ✓ Kept in a bowl of water. a) yes b) no
- ✓ Kept inside the sink without a running water supply. a) yes b) no
- ✓ Kept in a tray/bowl without water. a) yes b) no
- ✓ Used microwave and chiller. a) yes b) no

Food Handling Practices

- ✓ Use of gloves when handling ready-to-eat foods. a) yes b) no
- ✓ Avoiding bare-hand contact with food. a) yes b) no
- ✓ Proper covering and storage of leftover food. a) yes b) no

Food Preparation

- Use of separate cutting boards for raw meats and other foods. a) yes b) no
- Proper cooking temperatures reached and maintained. a) yes b) no
- Thawing of frozen foods done safely (e.g., not at room temperature). a) yes b) no
- Reuse of same oil in cooking food. a) yes b) no

Food Storage

- Cold at <5°C. a) yes b) no
- Freeze at < -18°C. a) yes b) no
- Hot food above 60°C. a) yes b) no
- Raw and cooked food is stored separately in the refrigerator. a) yes b) no

Food Display and Service:

- Proper covering and protection of displayed food items to prevent contamination. a) yes b) no
- Regular cleaning and sanitizing of display cases, buffets, and serving utensils. a) yes b) no
- Regular checking of expiry date of food items and properly dispose the expired food items. a) yes b) no

Wipe cloths

- Cloth dirty/bad-smelling. a) yes b) no
- Same cloths used. a) yes b) no

Cleaning and Sanitizing

- ✓ Regular cleaning schedules for equipment, utensils, and surfaces. a) yes b) no
- ✓ Use of approved sanitizers for disinfecting surfaces. a) yes b) no

Pest Control

- ✓ No signs of pests such as rodents, insects, or birds. a) yes b) no
- ✓ Proper storage of food to prevent pest access. a) yes b) no

Waste Management:

- ✓ Proper disposal of waste, including food scraps and packaging. a) yes b) no
- ✓ Adequate trash bins with lids to prevent contamination and odor. a) yes b) no
- ✓ Regular removal of waste to prevent buildup. a) yes b) no
